# Health system challenges in delivering maternal health care: evidence from a poor urban neighbourhood in South India

**DOI:** 10.1186/1753-6561-6-S5-P13

**Published:** 2012-09-28

**Authors:** BS Thriveni, Upendra Bhojani, Arima Mishra, Amruthavalli, Roopa Devadasan, CM Munegowda

**Affiliations:** 1 Institute of Public Health Bangalore, India; 2 Institute of Tropical Medicine, Antwerp, Belgium

## Introduction

India is committed to achieve the Millennium Development Goals to control maternal mortality (MDG 5). Such commitment is concretised in the launching of the National Rural Health Mission, which aims at improving access to quality health care for poor women and children living in rural areas. Despite evidence on rapid urbanisation and the absence of any health safety net for the urban poor, the proposed National Urban Health Mission (NUHM) remains in draft form. Drawing on evidence from a poor urban neighbourhood in Karnataka, the paper discusses the health system challenges for delivery quality maternal health care.

## Methods

The paper draws on fieldwork conducted in KG Halli, a poor urban slum neighbourhood in Bangalore. The fieldwork is part of a larger urban health action research project (UHARP), carried out in this area since 2009. The project aims at enhancing quality of health care of residents in this area. This paper is based on data collected over a period of two years (2009-2011) through (1) household census (n = 9,299, response rate = 98.5%) using a questionnaire on socio-demographic characteristics, illness profile, health seeking behaviour, and healthcare expenditure; (2) interviews with healthcare providers (n = 16), and (3) observational field notes on issues related to maternal health including mapping of health facilities in the area. Data were analysed from the lens of a health system analysis framework developed by Van Olmen and colleagues [1] to identify larger systemic challenges in delivery of service.

## Results

The mapping exercise shows that there are two government facilities and two private clinics providing antenatal care (ANC) and two private hospitals providing ANC and institutional delivery services for a population of over 44,500 in KG Halli. There is poor operational and administrative integration between different levels of care and facilities. A pregnant lady usually gets antenatal check-up at first-level care and delivers at maternity home (secondary level) or in a hospital at tertiary level. Referral to secondary or tertiary facilities is often based on informal understanding of rules among providers regarding registered ANC versus non-registered referred cases; the former determines the legitimacy for treatment in the secondary centre; secondary/tertiary government facilities that cater to most of the institutional deliveries are situated at a distance of around 6 km to 15 km from the area. Despite the distance and ‘patient-unfriendly’ attitude of the health staff, women deliver in government centres (56% of total institutional deliveries). This is due to both perceived high cost of care in private sector and apprehension that cost and number of caesarean sections performed is high in private sector. Our survey results show that 60% of all caesarean sections were conducted in private hospital, whereas 40% in government hospitals during the study period. Women reported that the cost of a caesarean section in a private facility is five times more than in the government facility. We also found that oversight mechanisms to ensure quality of care in both government and private facilities are poorly implemented as KG Halli lies on the border of two administrative sub-divisions and struggles to find a space between the ‘rural’ and the ‘urban’ administration.

## Discussion

Analysis of health service delivery in KG Halli from a health systems perspective identifies several constraints resulting in poor quality maternal health care. These systematic constraints need to be redressed at many levels to improve delivery of patient-centred maternal care. Such efforts would go a long way in not merely reducing maternal mortality ratio but also ensuring a robust local health system to deliver quality health care.

## Competing interests

All authors except the third author, are part of the team implementing the Urban Health Action Research Project in KG Halli, Bangalore

## Funding statement

The UHARP is funded by the Sir Dorabaji Tata Trust, MISEREOR, and by the Directorate-General for Development Cooperation and Humanitarian Aid (DGD), Government of Belgium, through the institutional collaboration between the Institute of Public Health, Bangalore and the Institute of Tropical Medicine, Antwerp and Medico International, Germany.
